# Behind the Silence of the Professional Classroom in Universities: Formation of Cognition-Practice Separation among University Students—A Grounded Theory Study in China

**DOI:** 10.3390/ijerph192114286

**Published:** 2022-11-01

**Authors:** Fenghua Xu, Yanru Yang, Junyuan Chen, A-Xing Zhu

**Affiliations:** 1 School of Education, Central China Normal University, Wuhan 430079, China; 2 Jiangsu Center for Collaborative Innovation in Geographical Information Resource Development and Application, School of Geography, Nanjing Normal University, Nanjing 210023, China; 3 Key Laboratory of Virtual Geographic Environment, Ministry of Education, Nanjing Normal University, Nanjing 210023, China; 4 Department of Geography, University of Wisconsin-Madison, Madison, WI 53706, USA

**Keywords:** classroom silence, professional courses, Chinese university students, separation of cognition and practice, grounded theory

## Abstract

Classroom silence is a negative form of classroom performance that is particularly prominent in the Chinese learner population. Existing research has mainly explored the silence phenomenon among Chinese university students in two types of learning contexts: overseas university classrooms and foreign language classrooms at local universities, without focusing on the Chinese undergraduates’ reticence in courses mediated by native language at domestic universities. However, the last type is the most common habitat for Chinese university students’ learning in higher education. Therefore, a sample of Chinese undergraduates majoring in education (n = 394) was recruited to determine the mechanisms of silence formation in professional classrooms. This study was based on grounded theory and in-depth interviews, and the recorded material was processed using NVivo 12. After a series of steps including open coding, axial coding, selective coding, and theoretical saturation testing, the core feature of the phenomenon of silence in professional classrooms of Chinese university students majoring in education was found to be the separation of students’ cognition and speaking practice. Then, a theoretical model of the formation and development of the phenomenon of classroom silence in professional classrooms of these undergraduates was constructed. The study showed that these university students had professional perceptions of classroom silence and displayed strong opposition to it, but they continued to maintain silent classroom behavior under the combined influence of individual characteristics, classroom experience, and learning adjustment. Following this, implications for existing research and suggestions for future practice are discussed.

## 1. Introduction

University students’ classroom performance is an important parameter for predicting their learning and academic achievements [1,2,3,4]. As a negative form of classroom performance, classroom silence among university students is a behavioral manifestation in which no verbal interaction occurs during classroom learning [5]. This form of classroom performance often does not lead to better learning gains; on the contrary, it may significantly and negatively affect university students’ deep learning and thinking and indirectly affect their academic performance [6]. Numerous experimental and empirical studies showed that interactive learning benefitted university students. A qualitative study revealed that teacher-student interaction was one of the factors that improved university students’ learning outcomes [7]. There were also studies based on an empirical comparative perspective that demonstrated that the gains of interactive learning were significantly better than those of silent passive individual learning [8,9], although some experimental studies proved that there was no significant difference in the impact of interactive or traditional classrooms on undergraduates’ performance. However, interactive classrooms could make classroom instruction more efficient. In addition, interactive classrooms were more helpful than traditional classrooms in closing the scores gap between students, which was particularly beneficial for students with lower scores [10]. In higher education institutions worldwide, university classroom silence is particularly prominent in the Chinese learner population [11,12,13,14,15,16,17]. Therefore, the phenomenon of classroom silence and the challenge of improving Chinese university students’ classroom learning performance have received much attention from researchers and practitioners.

Regarding the classroom silence of Chinese university students, existing studies have shown strong interest in two types of contexts. The first type of research explored the silence of Chinese students in overseas university classrooms. These studies mainly investigated the Chinese students’ reticence in university classrooms overseas such as in New Zealand [18], in the United States [15,19,20], and in Australia [21], to interpret and understand the causation of undergraduates’ classroom silent behavior by students learning from teachers with different cultural backgrounds. For example, Wilkinson and Olliver-Gray [18] conducted an exploratory study of Chinese learners’ lack of participation in classroom discussions, which was a frequent problem in New Zealand universities. Based on the concept of “cultural learning encounters”, the study unpacked the different interpretations of non-participation and excessive speaking by New Zealand and Chinese students and highlighted the need to create a culturally collaborative teaching model in university classrooms with international students based on a comparison of three different forms of a teaching organization. Because the subjects of such studies were in cross-cultural contexts, the cultural sensitivity of researchers often tended to make them much more concerned with the role of cultural factors (especially Chinese and Western cultural differences) in the reticence of Chinese students in classroom settings. Therefore, the findings of such studies are of less relevance in explaining Chinese university students’ performance in classroom silence in local cultural contexts.

The second type of research investigated the silence of Chinese undergraduates in foreign language classrooms at Chinese universities and its influencing factors [13,14,17,22,23,24,25]. For example, Liu and Jackson [25] (pp. 119–137) investigated the performance of 93 non-English major first-year freshmen at a top Chinese university in the English classroom. Through viewing course videos, reflective diaries, and interviews, the researchers found that although students self-reported a strong willingness to speak, their actual speaking behavior was low. The reasons behind this were complex, including multi-dimensional factors such as language, culture, education, psychology, and personality, but among them, the lack of English language ability or students’ lack of confidence in their English proficiency was the most important constraint. He [22] (pp. 87–142) investigated 302 non-English majors from two Chinese universities on the phenomenon of learning anxiety in university English classes. From the questionnaire, a lack of vocabulary or background knowledge was the primary cause of anxiety and silence. From the interview, students listed 16 reasons for anxiety and silence, of which 1/4 were factors directly related to English language ability. In contrast to the first type of research, this type of research broke out of the confines of cultural centrism. However, as the language medium in the classroom was English, researchers tended to consider the lack of English language skills as the dominant cause of Chinese university students’ classroom silence. Focus was on factors such as lack of self-confidence, fear of classroom participation, and fear of losing face due to poor performance to explain Chinese learners’ silent performance in English classes at domestic universities [13,14]. Therefore, such research was constrained by classroom language contexts and could not well reflect the silence of Chinese university students in classroom situations where the native language was the medium of communication.

Obviously, the studies above mainly discussed the speech situation of Chinese undergraduates in foreign language-mediated classrooms, but the studies on overseas classrooms also involved cultural heterogeneity in addition to language barriers. However, for Chinese university students, classrooms at Chinese universities that use their mother tongue as the medium of communication are the more important learning environments. Considering this, some studies have begun to focus on the third classroom situation. For example, Zhang and McNamara [16] (pp. 146–147) studied students’ participation in mathematics and Chinese classes at Shandong University and found that a lack of classroom interaction between teachers and students was common [13,14]. Lv analyzed the types of students’ silence in general education classes at Nanjing University and the complex psychological factors behind them [6,26]. In addition, some studies have begun to explore strategies to improve such silence in the classroom [27,28,29]. However, compared with the first two types of research, the research in this area was still very weak, especially for in-depth understanding of the silencing mechanism of Chinese university students.

Two insights can be drawn from current studies: on the one hand, Chinese undergraduates’ classroom silence exists in a wide range of classroom contexts; on the other hand, there is some variability in the attribution of Chinese students’ classroom silence from studies based on different classroom contexts. This suggests that changes in context may bring about changes in silence factors, which reflects the need to focus on new contexts. Therefore, this study intends to explore in depth the native language-mediated classroom context at domestic universities, which has received less research attention but is the primary classroom learning environment for Chinese university students, to further verify whether classroom silence still exists among Chinese undergraduates in conditions free from cultural differences and language adaptation and if so, what are the features and reasons of silence in this situation? Answers to these questions will help us deepen the understanding of Chinese university students’ classroom reticence; that is, when the language and culture that have been summarized by present studies are no longer the main reasons, what are the key factors contributing to this phenomenon? Since the professional courses of non-foreign language majors in Chinese universities are the most extensive native language classroom situations, this study will focus on the phenomenon of classroom silence among Chinese university students in such situations, and further, select the education major courses as the specific research context. Professional courses for undergraduates majoring in education are the main site for these students to learn educational theories, knowledge, and skills. After one to two years of professional study, these students have already acquired the preliminary professional quality of education, have a certain professional judgment ability on “what is a good classroom”, and have strong sensitivity as well as reflection on educational issues. At the same time, most of these students are potential future teachers, and their classroom learning experiences at the undergraduate level have a profound impact on their subsequent classroom teaching practices. Since China entered the 21st century, curriculum reform has been continuously promoted. One of the trends in the reform is to highlight the dominant position of students and emphasize students’ classroom participation. For this reason, new classroom learning methods such as cooperative learning have also been proposed [30,31]. Therefore, investigating the current situation and causes of classroom silence in professional courses of university students majoring in education will not only help to expand the understanding of classroom silence among Chinese undergraduates, but also can partially predict whether these future teachers will grow to be enablers or hindrances to the curriculum reform, so that action can be taken as early as possible. But we know very little about it due to omissions in existing studies.

To sum up, the purpose of this study is to reveal the specific representation and formation mechanism of professional classroom silence of Chinese undergraduates’ majoring in education. Specifically, taking university students majoring in education at a normal university in China as the participants, the grounded theory method is used to explore the experience of silence in the professional class and the complex factors behind it, and to develop a corresponding substantive theoretical model, to expand and deepen the recognition of Chinese university students’ classroom silence. Due to the similarities of cultural backgrounds, the conclusions of this study will also be applicable to explain the phenomenon of classroom silence among Asian university students elsewhere. In addition, due to the particularity of the participants, the relevant findings of this study will also help to grasp the reasons for the success or failure of curriculum reform that advocates students’ classroom interaction, in terms of the contribution of teacher education.

## 2. Materials and Methods

**Figure 1:**
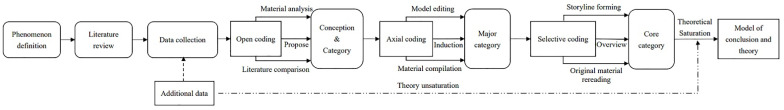
Flow chart of grounded theory.

This study uses a grounded theory approach to collect data, analyze information, and construct theory. Originally invented by Glaser and Strauss [32], the grounded theory approach was intended to oppose the deductive paradigm of using experience to generate theory. Grounded theory advocates for the discovery of theory from experience and then using theory to reflect the experience and serve an understanding of experience. The goal of grounded theory is to generate a theory from empirical material to explain a pattern of behavior that is relevant to the participant, or to the problem with which the participant is involved. Generally, such a theory is a substantive theory that is relevant, focused on individuality and complexity, and explores a particular phenomenon and its intrinsic connections. When the substantive theory accumulates to a certain extent it can also be developed into a more generalized formal theory. In general, grounded theory is a generative rather than a validated methodology [32] (pp. 2–3), and the flow of qualitative investigation using the grounded theory approach is shown in Figure 1.

We adopt the grounded theory approach commonly used in qualitative research to systematically collect and analyze empirical data for several reasons. First, since the purpose of this study is to explore the nature of silence in the professional classroom of Chinese university students majoring in education, a deductive-based approach is not applicable. Grounded theory is a proven method for exploring essentials, allowing concepts and categories to emerge naturally with greater objectivity, and has been successfully applied in many fields. For example, Burns and Schneider [33] used grounded theory to reveal the elements of leadership programs that had the greatest impact on the alumni’s lives and careers, as well as recommendations for how the program could better prepare students for the future. Second, the grounded theory approach is good at refining and summarizing the students’ learning experiences. Because grounded theory describes and conceptualizes respondents’ perspectives, behaviors, and lived experiences in the context of their lives, it ensures a participant-centered understanding [34] (pp. 131–146). Third, the nature and formation mechanism of classroom silence among Chinese undergraduates majoring in education have not been thoroughly studied, and grounded theory has important applications when theory and research are underdeveloped and underdefined [35] (pp. 5–7). Fourth, based on research in other contexts, the nature and mechanisms of silence in the classroom are complex and involve a variety of relevant factors, which is where grounded theory can be useful. Grounded theory is defined as “the discovery of theory from data systematically obtained and analyzed in social research” [32] (p. 1) and is a method that better reflects social psychological processes. The use of grounded theory as a methodology can help to reveal the formation mechanism of classroom silence among university students majoring in education in China.

### 2.1. Participants

Participants in this study were recruited by researchers from a normal university directly under the Ministry of Education in central China in spring 2019, spring 2020, and spring 2021. In China, normal universities are the main institutions for training future teachers. Education is the dominant discipline in normal universities, which generally cover both undergraduate and graduate levels; there are fewer comprehensive universities with education majors, among which, even fewer comprehensive universities have undergraduate education majors. Thus, the normal university was chosen as the study site. The university chosen on this basis is one of the six key comprehensive normal universities in China and one of the Chinese universities with a relatively high level of development of the discipline of education. Therefore, it is representative to choose this place for investigation. Considering that juniors have already had a long professional learning experience and have a deeper sense of classroom silence and that many seniors are not on campus due to internships or job searches, this study prefers to select junior students who have a professional course classroom silence experience. In March 2019, the researchers recruited 156 eligible participants by distributing recruitment leaflets. In March 2020, due to the impact of the epidemic, the researchers recruited 131 eligible participants by distributing electronic recruitment leaflets. In March 2021, the researchers recruited 107 eligible participants by distributing recruitment leaflets. In doing so, a sample of 394 participants was formed (see Table 1 for details). The inclusion criteria for participants were as follows: (1) have been living and receiving education in China; (2) are enrolled in an undergraduate program; (3) are majoring in education; and (4) have been identified by self-report and classroom instructor as having silent behavior in the classroom.

### 2.2. Procedure

**Figure 2:**
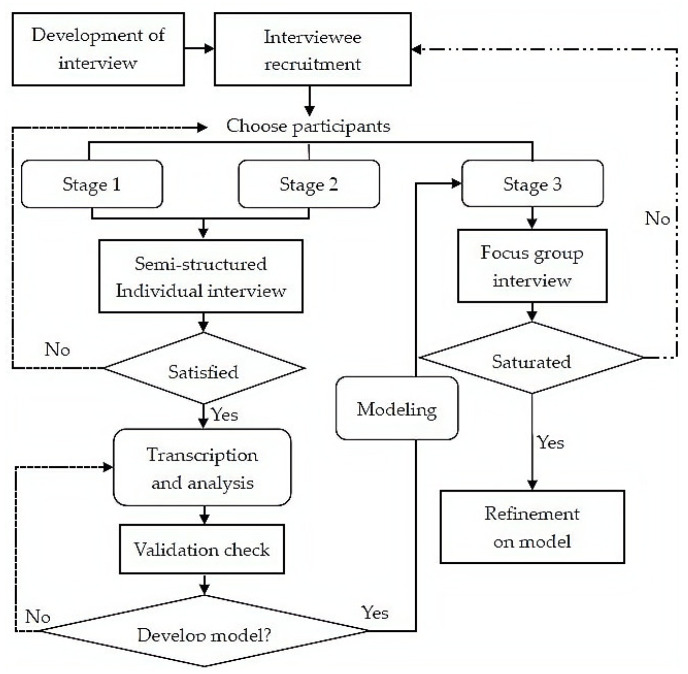
Flowchart of data collection and analysis based on grounded theory.

The grounded theory research procedure is characterized by the integrated nature of data collection and data analysis. Therefore, the data collection for this study was based on the principles of theoretical sampling [36] (p. 197). It adopted a strategy of mutual facilitation and dynamic generation and was conducted in three stages with semi-structured interviews. Theoretical sampling is a sampling that builds on concepts that have proven theoretical relevance in the developing theory [36] (p. 197). The principle of sampling is to keep adding to the sample as needed for theory development until each category in the data reaches theoretical saturation (i.e., no new theoretical elements emerge) [32] (pp. 61–62). Based on the informed consent of the interviewees, the researchers recorded the entire interview and transcribed it verbatim. Each interview lasted between 30 and 60 min. To ensure the privacy of the interviewees, we treated all interviewees anonymously. At the same time, the study tried to maintain rigor while processing qualitative data with the guidance of grounded theory [37,38] (see Figure 2 for details).

#### 2.2.1. Stage 1

This stage focused on building a preliminary interview system and coding system. First, an initial interview outline was prepared based on the purpose of the study (see Table 2). Based on this, the researchers selected 87 participants from the 156 participants recruited in March 2019 as a preliminary sample, including 5 freshmen, 18 sophomores, 57 juniors, and 7 seniors. These students not only had classroom silence in their professional classes but also had many views about it, through which they could help the researchers to better identify the characteristics of classroom silence and students’ attitudes in education major classes. Researchers conducted one-on-one semi-structured interviews with the participants. The interview locations were set in empty classrooms or the school cafeteria according to the interviewee’s preference. Although the researchers designed the interview outline in advance, the actual interview was followed up when the researchers found new and more valuable information at the right time. The interview questions were based on the interview outline and focused on the current state of silence in the professional classes experienced by the participants and the reasons for their silence. Starting with an understanding of classroom silence, the participants were allowed to share autobiographical accounts of themselves and their experiences in class [39] (pp. 119–121). Questions were then directed to the respondents’ perceptions and attributions of the silencing phenomenon in the professional classroom, strategies to improve it, and to pursue new questions that arose. Once all interviews were completed, two researchers simultaneously coded these 87 interview texts for independent analysis, and then the four researchers discussed the coding rationale and further interview questions together. To enrich the prototype coding system, the researchers continued to select 57 participants from the remaining participants for one-on-one semi-structured interviews and repeated the coding procedure above. This round of interviews further uncovered the reasons for the participants’ classroom silence. Subsequently, researchers conducted a focus group interview with the remaining 12 participants to confirm the developed coding system. To avoid participants’ nervousness, the researchers chose a smart classroom with a cozy environment and tables and chairs that could swing freely, arranged the tables and chairs in a ring shape in advance, informed and invited 12 participants to come to the interview at a uniform time, with Researcher 2 and Researcher 3 acting as the moderator and recorder, respectively. Questions were set up mainly around the interview outline and the results of the previous two rounds of interviews and were adjusted according to the on-site conversation, for example, asking the participants to talk about their perceptions of professional classroom silence based on their professional knowledge and experience to obtain more information for the study. The fieldwork was organized in a way that allowed data collection and preliminary analysis to occur simultaneously [40] (pp. 64–69).

#### 2.2.2. Stage 2

This stage focused on further validation and refinement of the coding system developed in the first phase. Only online interviews were used in this phase because students were learning online due to the epidemic. 78 participants were randomly selected from the 131 participants recruited in March 2020, and one-to-one semi-structured interviews were still conducted by the researchers with the participants. After that, the researchers selected 43 participants for one-on-one in-depth interviews and conducted a focus group interview for the remaining 10 participants. The specific process was similar to the first stage and will not be repeated here. This round of interviews was dedicated to fulfilling the attributes and dimensions of the emerged categories on the one hand, and to discovering new elements on the other [41] (pp. 36–54). After this round of interviews, four researchers continued to follow the first phase of independent coding followed by a consultative workshop. At this stage, the existing categories were further enriched. The relationship between these categories was clarified through the paradigm model of axial coding. Based on this, the researchers established the core category through selective coding and formed a theoretical model about the formation and development mechanism of the phenomenon of silence in the professional courses of Chinese undergraduates majoring in education.

#### 2.2.3. Stage 3

This phase focused on confirming or revising the theoretical model developed in phase 2. 52 of the 107 subjects recruited in March 2021 were selected by the researchers for one-on-one semi-structured interviews. The remaining 55 participants were divided equally into five separate groups for focus group interviews. The focus group interviews were conducted in empty classrooms at the university, and each focus group interview consisted of 2 researchers and 11 participants, one researcher was responsible for in-depth interaction with the 11 participants, and the other researcher was recording the interview. After this round of interviews, researchers found that no new categories or attributes emerged, and the theoretical model of the representation and formation mechanism of the phenomenon of the professional classroom silence of Chinese undergraduates majoring in education developed in the second round was further confirmed here. This meant that this study had achieved theoretical saturation at this point, and no new information needed to be collected [36,41].

### 2.3. Data Analysis

Coding is the key to generating theory from empirical data and thus is the core strategy for data analysis in this study. The “open coding-axial coding-selective coding” strategy invented by Strauss and Corbin [36] is widely accepted. In this study, this coding strategy was chosen and the interview data were analyzed level by level with the help of Nvivo12 software (QSR International, Burlington, MA, USA).

Open coding occurred mainly in the first stage of data collection and analysis and was centered on decomposing, comparing, labeling, conceptualizing, and categorizing data through line-by-line analysis. For example, the label “attitude” was assigned to the statement “Silence in the classroom has become a common phenomenon in professional classrooms nowadays, and for me, this is a bad phenomenon”. With the emergence of the new label “harm” from the supplementary interview, a new related concept “subjective perception” could be created on top of the two and further developed into the category “silence cognition”. Through this inductive and continuous comparison approach, five major categories were established at this stage. The axial coding occurred mainly in the second phase of data collection and analysis, which centered on the establishment of a paradigm model and the determination of the relationships among the major categories. A paradigm model is an analytical model for linking categories and sub-categories in a set of relationships at a higher level of abstraction [42]. In this study, the analysis of the five categories’ relationships clarified the status of the main category “classroom silence”. The core of the selective coding was to write a story line, select the core category, and complete the theoretical construction of the specific representation of classroom silence and its formation mechanisms among Chinese education major undergraduates. The selective coding was initially achieved in the second phase of the research process, and the rationality and credibility of the coding were further verified in the third stage.

### 2.4. Theoretical Saturation Test

The grounded theory approach requires researchers to continuously collect and analyze data and to continuously supplement and improve emerging concepts and categories [38]. When the newly collected data cannot be classified in new ways, this indicates that the theory has reached saturation [43]. After the first phase was completed, we organized the second phase of data collection and analysis to confirm whether new categories or concepts would be generated and found that new concepts and categories were indeed generated in the second phase, for which we organized the third phase. The third phase generated no new theoretical elements and further validated the previously coded logical relationships. This indicated that the previously constructed theoretical model was saturated. In addition, we fed back the categories and models generated by the coding to the professional course instructors and some of the interviewees. They confirmed that the model was consistent with reality and no more new categories were needed. The collection of new data was stopped because of the clarity and robustness of the extracted major class genera, initial class genera, and relationship descriptions. 

### 2.5. Rigor

The credibility of research starts from the data. The depth and scope of the data are very important. Research that generates data with rich area of coverage, rich content, and relevant information is exceptional [44] (pp. 24–25). In this sense, the larger the sample size of a qualitative study, the more representative the analysis of the sample will be of the population. However, the qualitative research process is more complex and depth-oriented, and it is not possible to select a very large sample as in quantitative research, so the sample size of common grounded theory studies is usually less than 100 [33,42]. Thus, it is clear that this study is rich and substantial in terms of both sample size and interview data while following the principles of theoretical sampling. In addition, it is particularly important to consider how to enhance self-reflexivity throughout the study [45]. To this end, the researchers used memos and reflections. After each interview and during data analysis, the researchers wrote memos about interview elements or illuminating data based on professional experience. At the same time, the researchers considered the possible impact of these ideas on the interview and analysis process through workshops and reflections and re-evaluated the process of interviewing and analyzing the data.

## 3. Results and Theory

Through theoretical sampling and a rigorous three-level coding procedure, this study focused on the basic situation of silence in the professional classroom of Chinese undergraduates majoring in education, and the real perceptions and explanations of students involved in the silence in the professional classroom. Responses to these questions formed the basis of the grounded theory of the formation and development of classroom silence within the professional course context. This section will use the three-level coding as providing clues to drive the analysis deeper layer by layer until the theory is generated.

### 3.1. Open Coding

The purpose of open coding is to develop a large number of codes to describe, name, or classify events [32] (pp. 35–39). Through fine-grained coding of the interview texts, in the open coding phase we grouped all data into 13 initial concepts: subjective awareness, objective awareness, speaking situation, class concentration, self-confidence, personality, speaking mindset, course attractiveness, peer influence, interaction convenience, educational experience, university environment and learning motivation. After further comparative analysis, a total of five major categories were extracted: silence cognition, silent behavior, personality characteristics, classroom experience, and learning adjustment (see Table 3). Detailed information about these categories and concepts will be specified in Section 3.3 of this study.

### 3.2. Axial Coding

**Figure 3:**
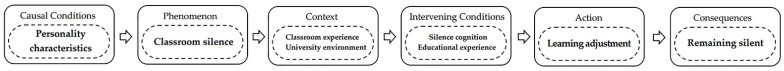
Schematic diagram of the axial coding of Chinese university students’ classroom silence.

Axial coding is the use of a combination of inductive and deductive reasoning to connect codes [46]. Following Strauss and Corbin’s paradigm model, this paper regrouped categories and attributes from the open coding. By analyzing the causal conditions, phenomenon, context, intervening conditions, action/interaction, and consequences of the phenomenon, the major categories and sub-categories were distinguished. Figure 3 showed the results of the axial coding.

As can be seen from Figure 3, “classroom silence” is the main category of the study, and the open coding of silence cognition, silent behavior, personality characteristics, classroom experience, and learning adjustment are all sub-categories for further understanding and interpretation of this main category. Through the paradigm model, the story context of silence in education professional courses among Chinese undergraduates becomes clear: the phenomenon of silence in education courses (the phenomenon) emerges among students majoring in education triggered by personality psychological characteristics such as introversion and fear (causal conditions). In turn, under the external conditions of inconvenient interaction, peer pressure, and a “strict entry and loose exit”, free and relaxing university environment (situational conditions), Chinese education major students adopt learning adjustment strategies such as lowering self-requirement or maintaining passive learning habits (action strategies), mediated by students’ silence perceptions and basic education experiences (intervention conditions), ultimately leading to the continuation of silent behaviors in education professional courses (outcome).

### 3.3. Selective Coding

Selective coding is the process of systematically selecting categories to find the core category by exploring the deeper relationships among the main categories. This paper described the relationship of each sequence using selective coding, focusing on the storyline “reasons for classroom silence in professional courses of university students majoring in education”. This section begins with a detailed description of the meaning of each category and the sub-categories it contains and cites the original data in which they are grounded when necessary.

#### 3.3.1. Silence Cognition

Silence cognition was the subjective awareness and objective cognition of Chinese undergraduates majoring in education about the phenomenon of silence in the professional classroom. It mainly included two categories of status quo perception and subjective cognition.

Subjective awareness

This referred to the participants’ perception of silence in the professional classroom and contained three aspects: the participants’ attitude, judgment on the nature of classroom silence, and the impact analysis. Firstly, almost all participants had a negative attitude toward silence in the professional classroom; they believed that silence in the professional classroom was “a very bad phenomenon” (P13, P27, P131) and a “persistent problem” (P16, P168) in the university classroom, and expressed their “disagreement” (P15, P97) with it. Secondly, one of the basic judgments given by the participants about the nature of classroom silence was that classroom silence was the opposite of classroom dialogue which alienated the potential two-way constructive activity in which both teachers and students participated into “a one-way activity” (P159, P201) on the teacher’s side, with no place for the students’ subjectivity to manifest. Finally, the participants provided a professional analysis of the harm of classroom silence. Participating students pointed out that this phenomenon not only limited multiple aspects of their development but also led to “a vicious circle” (P3) in which teachers lost the enthusiasm to teach and students lost the desire to learn.

Objective perception

This category was concerned with the objective description of the current situation of classroom silence by the participants. According to the general descriptions of the frequency, extent, and scope of classroom silence in professional classes by the interviewees, classroom silence was frequent and serious in such courses. “Classroom silence has become a normal for university students’ classroom learning.” (P42)

#### 3.3.2. Silent Behavior

Silent behavior was a silent activity of non-participation exhibited by students in the classroom, as evidenced by circumstances of speaking up and concentration on the course.

Speaking situation

According to the descriptions of the participants, the positive and initiative level in classroom interaction among university students today was not high. “Contemporary university students are always poorly motivated or engaged in the university classroom” (P27), which was a deep obstacle to classroom interaction. Even when there was a break in classroom silence, it was a passive choice forced by the final assessment. “Some students’ classroom participation would be significantly higher if the instructor made it clear that classroom presentations would be recorded in the overall final grade” (P24). 

Course concentration

Students’ attention was not focused on the classroom, and they appeared to “sleep” (P46), “indulge in temptations” (P9, P25, P40, P42, P78), “wander” (P43), and “do other assignments” (P12). Modern technological products made learning activities “a little bit tedious and long” (P39) and created a great temptation for students who lacked a clear goal and a strong will. Indulging in various digital temptations had become a prominent manifestation of silence in the university classroom (P4).

#### 3.3.3. Personality Characteristics

Students’ personality traits were individual factors of their silence in professional classes and its perpetuation. The results of the interviews and analyses showed that students’ silent orientation was closely related to their self-confidence, disposition, and speaking mentality.

Self-confidence

This study found that students’ self-confidence had a profound effect on their silent behavior in the classroom. First, a lack of self-efficacy caused students to lose the courage to answer questions. Students were often afraid to be the first speaker, “because if they don’t speak well, they will look reckless and stupid.” (P30) Second, speaking competency influenced students’ decisions about whether to speak in class. Many students “worry that they will not be able to give the right answer” (P1, P12, P23, P87, P167) because they “lack effective preparation” or “lack the ability to organize their thoughts and language in a short time” (P34); thus, they chose not to respond.

Disposition

According to the descriptions of the participating subjects, there was an important connection between individual students’ disposition and their classroom performance. First, introverted students had a natural tendency to remain silent. “For students who are introverted or timid, silence may happen on any occasion.” (P5, P11, P14, P20, P34, P73). Second, students who emphasized modesty and self-esteem would not express or present themselves in front of groups either. “Keeping face and remaining a low profile as well as humble attitude will reduce the frequency they speak or show in public” (P32). It is important to note that the determination of personality in this study was based on the self-reports of the participants and combined with the descriptions of the classroom instructors as well as the daily observations of the researchers. This is consistent with the basic requirements of grounded theory and has a basis in reality.

Speaking mentality

This was a factor that was relatively controllable by the individuals themselves and included fear, willingness to speak, and speaking needs. Most of the participating subjects expressed their fear of speaking in class, which could be categorized into three specific situations: fear of speaking, fear of being criticized by the teacher, and fear of being ridiculed or excluded by their classmates. Students might be afraid to speak up because they were “afraid of being blamed and criticized by the teacher for saying something wrong” or “afraid of being ridiculed by their classmates for incorrect comments” (P25). Another direct cause of classroom silence, as noted by some of the participants, was the lack of willingness to participate in class. “Some university teachers just read the content from a PowerPoint, causing students to lose interest in interacting with the teacher” (P13). Some of the participants indicated that speaking was unnecessary for classroom learning, so there was “no need for speaking” (P37).

#### 3.3.4. Classroom Experience

The classroom experience was the subjective feelings of the participating subjects about classroom learning in professional courses and the contextual factors of classroom silence in professional courses, which mainly included three sub-categories of course attractiveness, peer influence, and interaction convenience.

Course attractiveness

Course attractiveness often determined the extent to which students were willing to engage in the course, and it could be reflected in the teaching situation, student-teacher relationships, personal attributes of the instructor, score incentive, and classroom interest. According to the narratives of the participants, students did not participate in classroom interactions when the content was outdated, boring, or too abstruse (n = 67), the teaching style was single (n = 43), questions from the teacher were too difficult, too empty or too grand (P18, P41), “there is rejection written all over the teacher’s face” (P40), the teacher-student relationship was indifferent (P193, P275), there was a lack of score incentives (P30, P40), and they were not interested in the course (P4, P11, P83, P191).

Peer influence

The classroom was a collective, and students’ performance was often influenced and even pressured by other students. “When it becomes clear that no one is interacting with the teacher, students are reluctant to be the first to communicate for fear of being alienated by group” (P28). Another scenario of peer influence was if the number of students who were afraid to express their inner thoughts increased in the classroom, it would create a more serious classroom atmosphere. Those who were usually willing to speak were more likely to remain silent due to the collective silence of the majority, which was called “contagious silence” (P15, P24, P31, P77).

Interaction convenience

The ease of interaction reflected how easy or convenient it was for students to interact with the instructor and was primarily influenced by seating distribution, class size, and classroom equipment. The teacher-student relationship was alienated by the unreasonable layout of seating space, and “the general classroom seating arrangement does not facilitate discussion between the teacher and students and overemphasizes the teacher’s authoritative position” (P162). In addition, the “excessive class size” and “poorly equipped instructional equipment” (P32) also impacted students’ participation in the classroom.

#### 3.3.5. Learning Adjustment

Learning adjustment reflected the psychological and behavioral transformation of students from basic education featuring “high-intensity pressure” to higher education featuring “freedom and ease”. It was another important realistic factor related to the subjects’ professional classroom silence, mainly in terms of educational experience, university environment, and learning motivation.

Educational experience

Educational experience referred to the schooling experience of the participating subjects before they entered university, and was a historical factor that included educational inertia, study habits, and authority awareness. Most of the participants (n = 47) indicated that silence in class began to appear in primary and secondary schools, and gradually intensified with the increase of school years. The long-standing “indoctrination’ teaching” (n = 69), “exam-oriented education” (P2, P6, P30, P36, P73, P161), and “high-intensity pressure” (P8, P16, P211, P313) learning atmosphere had cultivated a learning habit of passive acceptance for students and fostered thinking inertia. The image of authority established by teachers in basic education also led students to “be afraid to show their differences” (P35). Therefore, the silence of the professional classroom in higher education was a natural extension of the silence of the basic education classroom.

University environment

Universities were the learning environment and living space of university students, and the management system and learning atmosphere of universities were the realistic factors for the silence in professional courses of the participants. On the one hand, the entry and exit mechanism and assessment system of universities inhibited students’ enthusiasm for self-expression. The “strict entry and lenient exit” (n = 45), together with the result-oriented and quantity-oriented assessment system (n = 27) of universities gave university students the capital to ignore the classroom (P8, P10, P93, P173). On the other hand, the free and undisciplined learning atmosphere of universities made it easy for students to let themselves go. Excessive free time (P24) and loose requirements from parents and teachers (P16) made students free to indulge in their respective worlds and appear silent in the classroom.

Learning motivation

The majority of participants (n = 63) reported that, due to the stark differences between the two educational systems, their demands for themselves and the emphasis on learning generally decreased after they entered university. Most students adjusted their learning goals from high scores to not “failing the exam” (P8). With the lowering of learning goals, students’ enthusiasm for classroom participation gradually disappeared, and thus, classroom silence happened. University students in a relaxed environment had a lax learning mindset (P10, P17, P38, P178), they were less committed to learning (P2, P21, P22, P41, P290), so they lost the prerequisite for classroom interaction. Considering this, the silence in class was “reasonable”.

From the analysis above, it was clear that the Chinese undergraduates majoring in education had a profound understanding of the nature and the harm of classroom silence in professional courses. At the same time, they perpetuated classroom silence that was barely recognized by themselves under the combined influence of multiple factors. As a result, the core category of the study emerged, namely, the separation of cognition and practice in the professional classroom silence of Chinese undergraduates majoring in education. Based on this, the researcher further clarified the attributes and dimensions of this core category (see Table 4).

**Figure 4:**
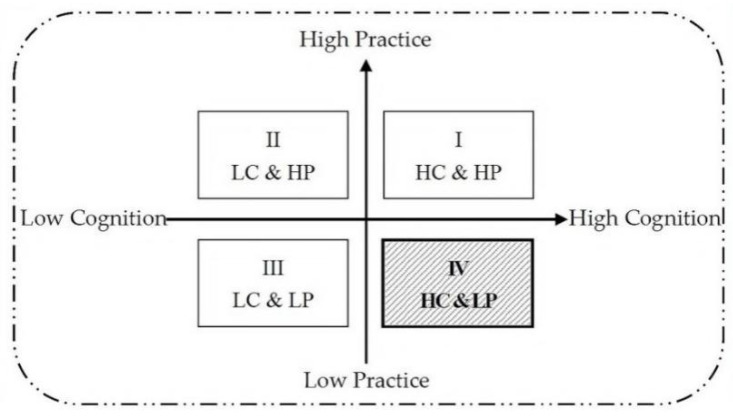
Quadrant diagram of the relationship between cognition and speaking practice of classroom silence.

Through in-depth analysis of the core category, the study found four variants of the undergraduates’ cognition and practice of classroom silence in their professional courses (see Figure 4).

### 3.4. The Theoretical Model of the Formation and Development of the Phenomenon of Professional Classroom Silence for Chinese Undergraduates Majoring in Education

**Figure 5:**
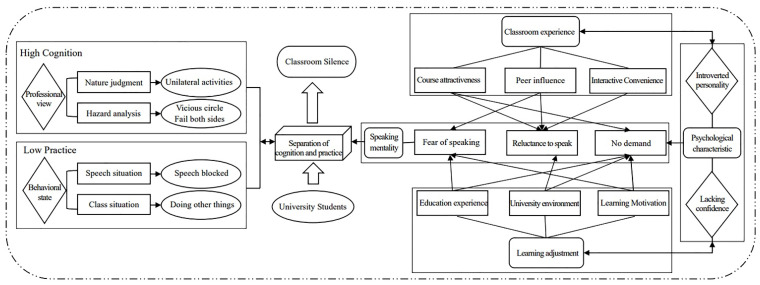
Model diagram of the formation and development of the cognition and practice separation in the classroom silence of Chinese undergraduates majoring in education.

The analysis above showed that the classroom silence of these students in professional classes featured the separation of cognition and speaking practice, which was manifested as “high cognition and low practice” (see Figure 4, quadrant IV), that was, students had professional cognition of silence’s hazards and disapproved of silence but remained silent practically. To deeply explain the mechanism of this phenomenon, this study constructed a theoretical model of the formation and development of cognition and practice separation in the classroom silence of Chinese undergraduates majoring in education. Figure 5 is the visualization of the formation mechanism of “high cognition, low practice”.

Chinese undergraduates majoring in education had a professional judgment about classroom silence due to their professional background. For example, they were able to recognize that students’ silent behavior in professional classes transformed teaching as a bilateral activity between teachers and students into a one-way activity on the teacher’s side and pointed out that the greatest harm was the formation of a vicious circle in which “teachers do not want to teach and students do not want to learn”. Through the interviews, we found that the judgment and reflection of education major students on classroom silence reflected strong professionalism, which was manifested in the terminology of expressions, such as “unilateral activity”, “alienation”, and “the sequela of basic education”. This is one of the characteristics that distinguish this study from other studies. Despite a high degree of professional awareness, they continued to behave as contributors to the phenomenon of silence in the professional classroom. This study found multiple causes for this phenomenon through an inductive analysis of data grounded in student interviews. The primary causes were students’ personality characteristics such as lack of confidence, introversion, and a mentality that hindered speaking. Among them, speaking mentality was the most direct cause of the silent behavior. The speaking mentality was not innate, some deeper causes could be further traced. For example, individual psychological characteristics of students, classroom experiences, the university environment, and the learning adjustments that occurred after students enter university. Some of these factors were situational, some were cultural, some were historical, and some were personalized. It was the interaction and joint influence of these factors that shaped students’ silent behavior in the classroom [47]. At the same time, this validated the paradigm model proposed at the phase of axial coding.

In other words, the speaking mentality was a direct cause of students maintaining silence in the professional classroom, and this relied on contexts such as the classroom experience and the university environment and it acted through mediating conditions such as silence cognition and educational experiences. Specifically, the fear of speaking was closely related to students’ psychological characteristics of lack of self-efficacy or introversion and was also rooted in their educational experiences, especially their personal experience of being criticized for wrong answers in basic education. Some students were afraid to speak because of their lack of speaking competency, which was closely related to their lack of knowledge reserves. The root cause was the loose study habits and negative study motivation of university students. The reluctance to speak was largely due to the students’ poor classroom experience, especially their low interest in course content or interactive topics. At the same time, peer pressure in the classroom experience might also weaken students’ willingness to speak for fear of being “embarrassed”, “perceived as being strangely” or “excluded”. This depended in part on the lax management and inappropriate evaluation of teachers and students in universities and also affected students’ adjustment to the university environment. The lack of need to speak was mainly due to the one-way input learning habits developed in the indoctrination classroom mode of basic education, which hindered the development of students’ thinking and expression skills. In other words, the inappropriate learning adjustment that occurred after students entered the relaxed environment of universities was the root cause of students’ lack of speaking needs.

The underlying causes were both relatively independent and interrelated. On the one hand, personality and psychological characteristics, classroom experience, and learning adjustment each constituted relatively independent influencing factors on the silence maintenance of the undergraduates in their major courses. On the other hand, the great contrast between the “high-intensity pressure” learning environment in basic education and the “free and easy” learning atmosphere in higher education generated learning adjustment behaviors, which interacted with their classroom experience through the influence of students’ psychological characteristics.

## 4. Discussion

### 4.1. Summary and Discussion

This study proposed a dynamic theoretical model of the formation and development of silence in the professional classroom of Chinese undergraduates with an education major by grounding in interview data. The main contribution of the study is to reveal the phenomenon of the separation of cognition and practice in the professional classroom silence among Chinese undergraduates majoring in education and to further investigate the mechanisms of this phenomenon. The main findings of this study will be discussed in further depth in comparison with related existing research.

First, this study found that not only the silence in the professional classroom was very common and serious among Chinese university students who majored in education, but there was also a separation of cognition and practice in which students had high cognition and low practice of speaking in the professional classroom (see Figure 4, quadrant IV). On the one hand, both the researchers’ informal classroom observations and the researchers’ in-depth interviews with the participants suggested that classroom silence was normal for university students. Silence in the classroom was still widespread among Chinese university students, even in professional classes at domestic universities where the native language was the medium of communication, which formed a mutually corroborating relationship with existing research on silence in overseas classrooms and second language classrooms in local universities [11,13,17,18,19,21,22]. On the other hand, all participants in the interviews had a negative attitude toward classroom reticence and made a more professional analysis of silence in the professional classroom, but at the same time took no measures to improve it, so that silence continued to occur or even gradually increased. Zhou [24] and Hsu [14] revealed in their study that there was a clear contradiction between Chinese university students’ perceptions of oral participation in university English classes and their actual behaviors. Although students valued classroom oral participation and did not wish to be passive learners, their overall level of participation remained low. Cheng’s [48] study focused on the reasons why some Asian ESL/EFL learners continued to fail to take an active role in the classroom although they might have a strong desire to speak. The research above identified some mutual evidence with the finding of this study, that there was a separation of cognition and practice among Chinese university students regarding classroom silence. In contrast, this study not only directly addressed Chinese university students’ perceptions of classroom silence but also explored the mechanisms that generated the cognition-practice gap of classroom reticence among Chinese university students in their native language classrooms at domestic universities.

Correspondingly, “low cognition, low practice” (see Figure 4, quadrant III) was observed in research on Asian students’ classroom reticence. Silence was a positive strategy for Asian students to save face and show respect and courtesy [49,50] as well as maintain harmony in the social order [51]. As a result, they often chose to be reticent for the sake of these positive functions of reticence both in domestic and foreign classrooms. A certain explanation of this interpretation difference might be that relevant studies tended to reveal the positive meaning of silence from the perspective of understanding silence or the cultural identity of the participants. While this study was not limited by a specific theoretical perspective (which was also a requirement of the grounded theory), due to the education professional background of participants, the analysis based on the interviews with these students inevitably reflected the disciplinary characteristics of education, i.e., showing the understanding of the essence of teaching and learning and paying more attention to the problems of education itself.

Second, the reasons why students remained silent in the classroom were systematic and diverse. This study found that students’ classroom silence in specific contexts was facilitated by a combination of factors. Many of these factors extended beyond the specific classroom context and were linked to the learning environment and its changes as well as the learners’ learning adaptations. Although the outwardly observable manifestations of silent behaviors such as students not taking initiative, not actively speaking, and doing things unrelated to the classroom were similar, the reasons behind them were not the same. Moreover, the combination of these factors could make students’ tendency to be silent more stable, making it difficult for students to induce changes in their actions even when they were highly aware of the dangers of classroom silence.

Relevant research had also explored the causes of students’ refusal to participate orally in class from multiple perspectives [5,13,14,25,52,53,54,55,56]. For example, King [5], from the perspective of dynamic system theory, pointed out that the state of silence prevalent in Japanese second language classrooms was built on many pillars. Even within a single lesson, there might be multiple interrelated reasons behind students’ silence. These reasons included both dynamically changing external factors as well as internal characteristics of learners. This systematic perspective was highly compatible with the model ultimately constructed in this study, with the difference that King’s [5] study used dynamic system theory as a prior research perspective, while this study generalized the findings by grounding them in the interview texts. In addition, this study did not stop at displaying the influential factors behind students’ persistently silent behavior in professional classes but further subdivided the factors into direct and deep causes. Then, the interrelationships among the causes were clarified to build a systematic hierarchy of the causes of persistent silence in professional classes among Chinese undergraduates majoring in education.

Finally, this study further confirmed some of the common factors that had been studied and also revealed some factors that had not received sufficient attention in relevant studies. For example, some of the influential factors summarized in this study, such as individual psychological factors such as self-confidence, personality, speaking mentality, and classroom contextual factors, were consistent with factors concluded from studies of Jia et al. [57], Wang et al. [58], Ai [21], Flowerdew and Miller [10], Eddy-U [13], Hsu [14], Liu and Littlewood [12], Sedova and Navratilova [59]. These factors constituted the general reasons for Chinese students’ silence in different types and contexts of university classrooms.

At the same time, this study also highlighted some new factors. This study found that the learning adjustment that occurred after students moved from basic education to higher education was both a cause of their professional classroom silence and a consolidating factor in their continued classroom silence despite their disapproval of silent behavior. These factors had not received the attention they deserved in related studies. As mentioned earlier, basic education not only helped students develop a passive acceptance of learning inertia, but its excessive emphasis on scores and standard answers also imposed limitations on the development of students’ thinking and expression skills. As one study revealed, Chinese students’ classroom silence was essentially a learned behavior, as they began to “learn to be silent” in elementary school [60]. Even in conversational contexts, teachers’ questions were usually not used to elicit reasoning or probe students’ understanding, but rather to check students’ memory [61], and unskilled questioning left students with very limited opportunities for inquiry and discussion in the classroom [57,62,63]. In the absence of any external intervention, these factors, which the participants called the “after-effects of basic education”, would accompany students to the university and became an important historical source of silence in the university professional classes. Most students entered Chinese universities with a change in their learning mindset and behavior as a result of changes in the learning environment, which manifested itself in the form of lowered attention to learning, relaxed self-requirement, and indulgence in external temptations. These factors were accompanied by a lack of knowledge and a scattered state of learning in the classroom, resulting in students losing the prerequisites for interaction in the dialogue situations of professional classes due to a lack of ideas or the absence of concentration.

The results of this study have important implications for countries or regions where university classroom silence also exists, such as Australia [50], the United Kingdom [58], and South Africa [64], and East Asian countries such as Japan and South Korea [12], which share a similar culture and educational context as China. Generally speaking, improving silent behavior in the classroom requires dealing with factors such as classroom experience and learning adaptation to achieve consistency in university students’ cognition and practice. First, educators should focus on fostering an encouraging, positive classroom atmosphere and arranging open-ended discussions to increase student friendship and teamwork which means a non-threatening environment. Second, educational administrators should pay attention to students’ adaptation problems during the educational phase transition and adopt a combination of centralized preaching and individual counseling to help students make a smooth transition. Third, students should be guided to continuously increase their knowledge base and keep the classroom progressively active. In the beginning, students are invited to express ideas as a group rather than as individuals; then they are encouraged to express ideas as individuals; eventually, they will be able to actively share their views.

### 4.2. Limitations

This study focused on students majoring in education, so the results obtained might be influenced by the professional attributes of the participants. The results of this study showed that the core feature of the silencing phenomenon in professional courses in Chinese universities was the separation of cognition and speaking practice among university students, which was characterized by “high cognition and low practice”. The “high cognition” might be related to the professionalism and sensitivity of such a student group. Due to the contextual limitations of the substantive theory of “cognition and practice separation of Chinese education undergraduates’ professional course silence”, whether the findings of this study can apply to those from different majors should be further discussed. Therefore, subsequent studies need to expand the professional dimension by conducting research on classroom silence in different types of professional courses to explore whether the “high cognition, low practice” (see Figure 4, quadrant IV) model of classroom silence formation and development can explain classroom silence for other majors outside of education in Chinese universities.

In terms of sampling, future research needs to include the classroom silence and participation of junior students in various categories of majors at representative comprehensive universities to develop or revise the findings of this study. From the perspective of seeking contextual variables to increase the density and explanatory power of the theory, future research should focus on whether there is a unity between cognition and practice of “high cognition, high practice” (see Figure 4, quadrant I) or “low cognition, low practice” (see Figure 4, quadrant III) in Chinese university students’ professional course learning and a new separation between cognition and practice of “low cognition, high practice” (see Figure 4, quadrant II).

Due to the use of a qualitative research method based on grounded theory, the findings obtained in this study lacked large-scale quantitative validation. Therefore, using quantitative research methods and statistical analysis to validate the findings of this study and further explore the effects of other factors such as age, gender, socioeconomic status, career prospects and academic achievement on Chinese university students’ silent behavior in the classroom are also our next directions.

## 5. Conclusions

To investigate the phenomenon of silence in the professional classroom among Chinese undergraduates, this study used a grounded theory approach to conduct in-depth interviews and coding analysis with 394 Chinese undergraduates majoring in education who had experiences of silence in the professional classroom. The study found that students maintained silence while holding negative attitudes toward the phenomenon of classroom silence in professional classes. This finding further reinforces the relevant findings of existing studies (see Section 4.1 for details). However, the outstanding contribution of this study is not only to show the existence of this phenomenon but also to reveal the formation mechanism of the cognition-practice mismatch of students’ silence in the professional classroom with the help of the grounded theory approach. In terms of high cognition of classroom silence, the students’ background in education gives them a certain professional advantage in analyzing educational and learning issues. This insight further influences their attitudes toward silent behavior in the professional classroom. In terms of the low practice of classroom speech, speaking mentality is the direct cause. In contrast, personality and psychological characteristics, classroom experiences, and learning adjustment as deeper causes jointly influence students’ speaking impediments, thus indirectly contributing to the persistence of silent behavior in students’ professional classes. In addition, this study provides some new insights based on reaffirming and deepening the relevant findings of existing studies. For example, this study finds that learning adjustment contributes equally importantly to Chinese university students’ classroom silence, but these factors have not received enough attention in existing research.

## Figures and Tables

**Table 1:** Sample distribution (total = 394).

Characteristic.	Classification	Numbers	Percentage
Gender	Female	273	69.29%
	Male	121	30.71%
Education Level	Freshman	33	8.37%
	Sophomore	57	14.47%
	Junior	278	70.56%
	Senior	26	6.6%
Major	Education	198	50.25%
	Preschool Education	56	14.21%
	Special Education	78	19.79%
	Educational Technology	33	8.38%
	Physical Education	29	7.37%

**Table 2:** Interview outline.

Number	Questions
1	Please tell us about the state of your professional classroom learning.
2	What is the overall state of classroom learning in your class?
3	How do you feel about your and your classmates’ silent behavior in class?
4	Why are you silent?
5	What steps do you think could be taken to improve classroom silence?

**Table 3:** Open coding table for professional course silence of Chinese university students.

Original Statements (Partially Listed)	Labeling	Initial Categories	Major Categories
P27: Silence in the university classroom has become a common occurrence in professional classrooms today, and to me, this is a bad phenomenon.	Attitude	Subjective awareness	Silence cognition
P159: Classroom dialogue does not happen and teaching becomes a one-way activity.	Nature		
P131: Silence in the classroom prevents students from learning better and also discourages teachers from teaching.	Hazard		
P14: There are often no students willing to take the initiative to answer questions from the teacher.	Silence frequency	Objective perception	
P7: This phenomenon is becoming increasingly common and serious in university classrooms, and the reasons are varied.	Silence degree		
P33: Classroom silence among university students is quite common.	Silence range		
P27: Contemporary university students are always poorly motivated and engaged in the classroom.	Speaking activeness	Speaking situation	Silent behavior
P24: Some students’ classroom participation will be significantly higher if the instructor makes it clear that classroom presentations will be recorded into the overall final grade.	Speaking motivation		
P9: I am not interested in the content taught, and I think I can pass the exam by casually learning.	Learn casually	Course concentration	
P78: They use these electronic devices to do things that are not related to the classroom.	Doing other assignments		
P87: Students are more likely to think about whether they can answer well and thus defend their self-esteem with silence.	Self-efficacy	Self-confidence	Personality characteristics
P1: I think I can’t answer correctly so I don’t respond.	Speaking competence		
P32: Some reduce the number of times they speak in public out of “saving face” or maintaining a low profile and modest attitude.	Modesty	Disposition	
P19: Students generally believe that speaking in class is a thing that “loses face”.	Lose face		
P73: Students who are introverted and reticent may be reluctant to actively speak in class.	Introvert		
P6: The fear of answering incorrectly is also a major reason that prevents students from speaking up.	Fearfulness	Speaking mentality	
P34: They are willing to think about the questions asked by the teacher but are not willing to take the initiative to answer them.	Willingness to speak		
P12: They believe that speaking is not necessary for learning,	Speaking needs		
P25: The lecture content format is not attractive and does not motivate students to answer questions.	Teaching situation	Course attractiveness	Classroom experience
P275: The teacher-student relationship is not harmonious, and students do not want to answer teachers’ questions.	Student-teacher relationship		
P42: In my opinion, the teachers in the more active classes are generally interesting.	Teacher’s attributes		
P30: If it weren’t for the “usual score” test in class, there would probably be no one in class.	Score incentive		
P37: Lack of interest is a very important reason leading to students’ unwillingness to speak.	Classroom interest		
P28: Students are reluctant to be the first to communicate and fear being alienated or rejected by the group.	Peer pressure	Peer influence	
P77: The classroom atmosphere is serious, and students are afraid to break the calmness and take the initiative to speak.	Classroom atmosphere		
P162: The classroom seating arrangement does not facilitate discussion between the teacher and students.	Seating distribution	Interactive convenience	
P32: On a physical level, first, large classes make it difficult to create effective interactions. Second, the equipment in the classroom does not support effective dialogue.	Class size		
	Equipment constraints		
P36: Exam-oriented education makes students accustomed to the classroom style of teacher lecturing.	Educational Inertia	Educational experience	Learning adjustment
P107: The long-time habit from elementary school to high school causes students to maintain their original listening habits and gradually develop classroom silence when they enter the university classroom.	Learning habits		
P35: Basic education establishes the image of teacher authority and knowledge authority, which leads students to be afraid to challenge teachers and textbooks.	Authority awareness		
P2: The strict entry and lenient exit of domestic universities also give students the capital to ignore the classroom.	Management system	University environment	
P24: The free and diffuse atmosphere of the university campus somewhat undermines students’ motivation to perform in class.	Learning atmosphere		
P8: Most students simply require that they do not fail the exams in each course.	Self-development	Learning motivation	
P2: Because of the different levels of attention, university students are generally scattered and not motivated to study, so it makes sense that they are silent in class.	Learning emphasis		

**Table 4:** Core category and refinement analysis.

Core Category	Attribute	Dimension
Separation of cognition and practice	Cognitive level	High–low
	Behavior state	Speaking–No speaking

## Data Availability

The data presented in this study are available on request from the corresponding author. The data are not publicly available due to privacy.
